# Extracurricular school-based sports as a motivating vehicle for sports participation in youth: a cross-sectional study

**DOI:** 10.1186/1479-5868-11-48

**Published:** 2014-04-07

**Authors:** An De Meester, Nathalie Aelterman, Greet Cardon, Ilse De Bourdeaudhuij, Leen Haerens

**Affiliations:** 1 Department of Movement and Sports Sciences, Ghent University, Watersportlaan 2, 9000 Ghent, Belgium; 2 The Policy Research Centre on Sports, funded by the Flemish Government, Brussels, Belgium; 3 Department of Developmental, Personality and Social Psychology, Ghent University, Watersportlaan 2, 9000 Ghent, Belgium; 4 The Flemish Research Foundation, Brussels, Belgium

**Keywords:** Extracurricular school-based sports, Community sports, Physical activity, Motivation, Self-determination theory

## Abstract

**Background:**

Extracurricular school-based sports are considered to be an ideal means of reaching children who are not active in community sports. The purposes of this study were to examine the extent to which pupils not engaging in community sports do participate in extracurricular school-based sports, and to assess whether extracurricular school-based sports participants are more physically active and/or more autonomously motivated towards sports in daily life than children who do not participate in extracurricular school-based sports.

**Methods:**

One thousand forty-nine children (53.7% boys; *M* age = 11.02 years, *SD* = 0.02) out of 60 classes from 30 Flemish elementary schools, with an extracurricular school-based sports offer, completed validated questionnaires to assess physical activity (Flemish Physical Activity Questionnaire) and motivation (Behavioral Regulations in Physical Education Questionnaire). Multilevel regression analyses were conducted to examine the data generated from these questionnaires.

**Results:**

More than three quarters of the children (76%) reported participating in extracurricular school-based sports during the current school year and 73% reported engaging in organized community sports. Almost two third of the children (65%) not participating in community sports stated that they did participate in extracurricular school-based sports. Extracurricular school-based sports participants were significantly more physically active than children not participating in extracurricular school-based sports (β = 157.62, *p* < 0.001). Significant three-way interactions (sex × extracurricular school-based sports participation × community sports participation) were found for autonomous motivation, with boys engaging in extracurricular school-based sports but not in community sports being significantly more autonomously motivated towards sports than boys not engaging in community or extracurricular school-based sports (β = 0.58, *p* = 0.003). Such differences were not noted among girls.

**Conclusions:**

If extracurricular school-based sports are offered at school, the vast majority of elementary school children participate. Although extracurricular school-based sports attract many children already engaging in community sports, they also reach almost two third of the children who do not participate in community sports but who might also be optimally motivated towards sports. As children participating in extracurricular school-based sports are more physically active than children who do not participate, extracurricular school-based sports participation can be considered to contribute to an active lifestyle for these participating children.

## Background

Research suggests that a considerable amount of children and adolescents drop out from sports when they grow older [1,2]. Consequently, by the end of elementary school a considerable amount of children does not meet the health-related recommendations of sixty minutes or more moderate to vigorous physical activity (MVPA) per day [3]. Sports are considered one specific form of PA that contributes to children’s and adolescents’ overall engagement in MVPA [4]. When compared to other forms of physical activities, sports participation typically involves physical exertion and skill development, and the competition of individuals or teams against one another [5]. In order for more children to meet the health-recommendations regarding PA, the need arises to develop and implement promotion strategies for an active lifestyle, with the promotion of sports participation among children and adolescents as one possible strategy.

Schools are considered to be ideal settings for implementing such promotion strategies since children of all socioeconomic backgrounds spend considerable amounts of time at school. Furthermore, while required educational training degrees differ between countries, in most countries teachers are required to have a teacher education degree and most school are equipped with the necessary facilities to provide PA opportunities [6-8]. Although Physical Education (PE) is acknowledged to be an important intra-curricular medium within the school context for the promotion of sports participation among youth [9,10], PE hours in most school curricula are limited, and in many countries not mandatory [11]. Furthermore, within the limited curricular time, physical education’s diverse learning objectives are not exclusively related to promoting PA and sports participation. Within the school-community context possible additional sources for PA opportunities, including sports participation, thus need to be explored [12].

Due to considerable differences between different educational systems, various terms are used interchangeably within the literature to describe possibilities for school-based sports activities outside the PE curriculum (extracurricular school-based sports). Most authors apply the term extracurricular school-based sports when referring to the provision of activities at school outside the formal PE curriculum, most often during after-school hours and lunch breaks [13-15]. In the current paper, extracurricular school-based sports are defined in a similar way: ‘all sports activities not included in the curriculum but organized by the school either during lunch break, during after-school hours or on Wednesday-afternoon (typically a free afternoon in Flemish elementary schools) in which pupils can voluntarily participate’.

Extracurricular school-based sports are considered to be easily accessible in terms of location and costs. They usually involve participation together with class- or schoolmates and are generally less competitive when compared to organized community sports which makes participation not limited to sports-talented children [16]. Hence, in such a context, extracurricular school-based sports programs are typically put forward as programs that reach those children who are not participating in community sports [17]. This is different to the situation in the US, where extracurricular school-based sports can sometimes be selective and highly competitive in nature [18]. Despite these positive inclusive features of extracurricular school-based sports, little research has been conducted to explore which children participate in extracurricular school-based sports programs, and whether such programs indeed reach children who are not active in community-based sports. Hence, the first aim of the present study was to investigate how many children not engaging in community sports do participate in extracurricular school-based sports programs.

There is already some evidence suggesting that the provision of PA organized at school is effective to increase children’s activity levels, with children being more physically active if there is an extensive PA-offer at school [19,20]. According to Powers et al. [14], pupils’ PA-levels at school could be improved by increasing the participation, duration, and frequency of existing programs and by implementing programs before school and at lunchtime. The potential of such programs for the promotion of PA among adolescents is also acknowledged by other authors [15,21,22]. However, in most of these studies, it is not clear whether certain groups of pupils, characterized by common demographic (sex, age, SES), psychosocial (e.g., motivation, social support) or behavioral (e.g., participation in community sports) characteristics are more or less represented in extracurricular school-based sports, and whether pupils participating in extracurricular school-based sports are overall more or less active than their non-participating counterparts. Pupils who participate in extracurricular school-based sports are physically active during the time spent in extracurricular school-based sports but it is not clear whether they are more or less physically active outside these extracurricular school-based sports. Consequently, a second purpose of the present study was to investigate whether the group of pupils participating in extracurricular school-based sports differs from other groups (not participating in any organized sports or participating in community sports) in terms of overall activity levels.

Besides differing in PA-levels, children participating in extracurricular school-based sports might also differ from those not participating in extracurricular school-based sports in their motivation towards sports. Specifically, some pupils might be optimally motivated toward sports, but not get the chances (e.g. no parental support) to participate in sports outside the school context [23]. For this group of pupils, participation in extracurricular school-based sports might provide the only extracurricular opportunity to be active. Hence, a third aim of the present study was to investigate differences in motivation towards sports between children participating in extracurricular school-based sports and children not engaging in any form of organized sports.

In the present study, the concept of motivation was approached from the perspective of the Self-Determination Theory (SDT) [24]. This macro-theory of motivation provides an understanding of why people initiate and persist in behaviors [25] and distinguishes between autonomous motivation, controlled motivation and amotivation for sports. Autonomous motivation involves the regulation of behavior with the experiences of volition, psychological freedom, and reflective self-endorsement and is considered the most optimal form of motivation [26]. The second type of motivation is controlled motivation, which refers to the pressured engagement in an activity. Autonomous motivation and controlled motivation are contrasted with amotivation, which exists when people lack intentionality or engage in behaviors for unknown reasons [24]. For example, an amotivated pupil claims to have no idea why he should bother participating in extracurricular school-based sports. Hagger et al. [27] examined the relation between motivation for PE and motivation for sports in leisure time, outside the school. However, the relationship between extracurricular school-based sports participation and motivation towards sports has, to our knowledge, not yet been investigated. If we find that extracurricular school-based sports programs mainly attract those children who are already autonomously motivated towards sports, and furthermore there remains a large group of children that is not optimally motivated and is also not reached, the question arises how the promoting role of extracurricular school-based sports can be optimized so that groups with less optimal motivational profiles are reached.

In summary, the primary aim of this study was to investigate how many pupils not engaging in community sports do participate in extracurricular school-based sports. The second aim was to assess if extracurricular school-based sports participants were more physically active in daily life than children who do not participate in extracurricular school-based sports, while controlling for their participation in community sports. The last aim was to evaluate whether children who participate in extracurricular school-based sports, compared to those who do not, are better (i.e., more autonomously) or relatively worse (i.e., more controlled) motivated or amotivated to participate in sports in general. As the transition from childhood to adolescence is known to be a risk-period for decreased PA and sports participation [28,29], the target group of the current study consisted of children of the last two grades of elementary school.

## Methods

### Participants and procedure

Principals of 35 elementary schools in Flanders (the Dutch-speaking northern part of Belgium) with an extracurricular school-based sports offer were contacted to participate in the present cross-sectional study. The schools were randomly selected from a list of all schools located in two Flemish provinces (West Flanders and East Flanders) that participate in an extracurricular school-based sports project developed by the Flemish School-sports Foundation and the Flemish Commissariat for Promotion of Physical Development, Sports and Outdoor Recreation. This project provides logistical support to the participating schools who, in return, organize at least one weekly hour of extracurricular school-based sports during after-school hours on a weekday under the guidance of a PE teacher. At the moment of data collection, one school no longer participated in the extracurricular school-based sports project and it therefore did not meet the inclusion criteria. In addition, four principals of the other contacted schools refused to participate. This resulted in a final sample of 30 schools, of which eleven (36.67%) were located in an urban area, fourteen in a suburban area (46.67%) and five in a rural area (16.67%). Two classes in each school were randomly selected to participate in the present study (one class in grade 5 and one class in grade 6).

The children in the selected classes and their parents received an information letter and with the exception of 33 (response rate = 96.95%), all parents gave informed consent for their child to participate. In total, 1049 children (563 boys; 53.67%) with a mean age of 11.02 years (*SD* = 0.02, range 9-13 years) filled out a set of questionnaires during a randomly chosen lesson. All the questions were read out loud by the first author and clarified when necessary.

In the present study extracurricular school-based sports were defined as ‘all sports not included in the curriculum but organized by the school either during lunch break, during after-school hours or on Wednesday-afternoon, in which pupils can voluntarily participate’. In Flanders, (Belgium) elementary school-hours are traditionally organized between 8.30am and 12pm and between 1pm and 4pm. On Wednesday, the lessons end at 12pm and the pupils are free in the afternoon. Many schools therefore organize activities (including extracurricular school-based sports) on Wednesday-afternoon in which pupils can participate on a voluntary basis. In Flanders, extracurricular school-based activities can be organized at school or in the schools’ neighborhood. Extracurricular school-based sports delivered in the school’s neighborhood are not substantively different from those delivered at school, they merely take place in a different location (e.g., adjacent grass fields or sports halls off school campus). Extracurricular school-based sports activities are also sometimes organized for groups of schools, rather than for one single school, for which then a central location (yet still close to each of the participating schools) is often chosen. Extracurricular school-based sports during the weekend were not included in our definition because Flemish extracurricular school-based sports are rarely or never organized during the weekend.

The study protocol was approved by the Ethical Committee of Ghent University.

### Measures

#### Demographic variables

Demographic variables such as sex, date of birth and residence were obtained through a number of questions at the beginning of the questionnaire. Pupils were also asked to fill out their parents’ occupations. The Four Factor Index of Social Status [30] was then used to determine the pupils’ socioeconomic status (SES). This index determines a person’s SES, based on three parental factors: marital status, educational factor and occupational factor. A fourth factor applies only to retired persons. Given the young age of the participants in the current study, we focused on the occupational factor of both parents. Parents’ occupations were given a score from 1 to 9 and accordingly categorized in two socio-economic groups: low and medium SES (54.72%) on the one hand and high SES (45.28%) on the other hand.

#### Self-reported physical activity

PA-levels were determined using the Flemish Physical Activity Questionnaire (FPAQ) [31]. The pen and paper version of the FPAQ has been shown to be a reliable (test-retest reliability coefficients ranging from 0.69 to 0.93) and valid (concurrent validity: r = 0.27-0.44 with respect to accelerometer data) instrument to assess different dimensions of usual PA and sedentary behavior in children [32]. To determine the validity of the questionnaire in the current study, we calculated correlations between self-reported PA levels and accelerometer-measured activities in a convenience sample of 61 children who filled out the FPAQ. Similar to previous studies, significant positive correlations (r = 0.311; p = 0.045) were found between PA levels and accelerometer-measured activities. An additional file contains a detailed methodology of the validity study in our subsample (see Additional file 1). For the present study a distinction was made between the assessment of extracurricular school-based sports and other types of physical activity. Questions with regard to extracurricular school-based sports were similar as in the study of Haerens et al. [33]. In a first question, the extracurricular school-based sports offer at school was addressed (“Which of the following extracurricular school-based sports does your school offer during the current school year?”). Pupils could indicate multiple answers in a list of 50 sports or fill out a sport that was not included on the list in the space provided. Pupils were then asked about their personal extracurricular school-based sports participation, and were asked to indicate ‘yes’ or ‘no’ as an answer to the question “Did you participate in extracurricular school-based sports in the current school year?”. Pupils were instructed to indicate ‘yes’ to this question if they had previously participated in extracurricular school-based sports within the ongoing school year or if they had registered for upcoming extracurricular school-based sports activities within the ongoing school year. Pupils were then questioned about the frequency of participation in extracurricular school-based sports within the current school year: “How many hours do you spend on extracurricular school-based sports (a) during lunch break, (b) during after-school hours, (c) on Wednesday-afternoon?”. Multiple-choice answers to each of the three sub-questions varied from less than 1 hour per month to more than 4 hours per week, with seven sub-categories in between. Children’s weekly time spent in extracurricular school-based sports was calculated based on the answers on the three above mentioned sub-questions. An additional question was added about the specific sports they personally participated in (“Which of the following extracurricular school-based sports did you participate in during the current school year?”). Pupils could, similar to the question about the extracurricular school-based sports offer, indicate multiple answers in a list of 50 sports or fill out a sport that was not included in the list. Finally, to broaden the window to multiple school years, pupils responded to the question “How long have you been participating in extracurricular school-based sports?” via a multiple-choice answer, ranging from 1 (this is the first school year) to 3 (more than 2 school years).

Next to the questioning on extracurricular school-based sports, three categories of physical activities were measured using validated questions [34]: a) Intra-curricular PA (e.g. “How many hours of PE do you participate in per week?”), b) walking and cycling (e.g. “How much time do you usually spend cycling on a weekday?”, not distinguishing between walking and cycling for active transportation and leisure time), and c) non-school based sports participation (both organized as well as unorganized sports). Sports participation was assessed by asking the participants to indicate the main sport(s) they engaged in (with a maximum of 3 sports). For each sport, children had to report the frequency, the usual time spent on that activity and whether or not they practiced their sport(s) in a sports club (that is to make the distinction between organized community sports versus unorganized sports) [35]. Sports participation did not include extracurricular school-based sports, which was clarified to the pupils by means of an oral explanation by the researcher and the written instructions in the questionnaire. Children’s overall weekly PA was calculated by making the sum of the time (in minutes) spent in extracurricular school-based sports, intra-curricular PA-time, time spent on walking and cycling, and time spent in (non-school based) sports participation.

#### Motivation towards sports

An adapted version of the Dutch Behavioral Regulations in Physical Education Questionnaire (BRPEQ) [36] was used to assess motivation towards sports. The BRPEQ was validated in previous research in the context of PE [36] and is an adapted version of the BREQ-2 [37]. To obtain an optimal balance in controlled motivation items, the BRPEQ contains 6 additional items compared to the BREQ-2. Two other adaptations were made: one item was reformulated in order to avoid a double negative and the word ‘personally’ was added in another item because SDT emphasizes personal endorsement rather than the more general importance of engagement in activities [36]. Similar to the original BREQ-2, the sports context (all forms of sports in leisure time, whether or not organized within the community) was used to frame the items of the BRPEQ used in the current study.

The questionnaire consists of twenty items. Sixteen items with the stem ‘I participate in sports because…’ were used to measure autonomous motivation (8 items, α = 0.87; e.g., “I participate in sports because I find it a pleasurable activity.”) and controlled motivation (8 items, α = 0.70; e.g., “I participate in sports because I feel guilty if I don’t.”). Amotivation was measured by four items (α = 0.77; e.g., “I don’t see why I should bother participating in sports.”). Participants responded to each of the twenty items via a 5-point Likert scale from 1 (*not at all true for me*) to 5 (*very true for me*).

### Analyses

SPSS (version 19.0) was used to describe the characteristics of the sample, to describe the percentage of pupils participating in extracurricular school-based sports that either did or did not engage in community sports and to analyze the sports offer in the extracurricular school-based sports programs. Pearson’s chi-squared tests were then used to determine the relationships between sex, extracurricular school-based sports participation and community sports participation. The hierarchical structure of the data, with 1049 children being nested within 60 classes nested within 30 different schools, and the adequate sample size for conducting multilevel analyses [38], allowed us to employ multilevel regression analyses to examine whether children participating in extracurricular school-based sports are (1) more physically active in daily life and (2) better motivated to participate in sports than children who do not participate in extracurricular school-based sports. The total weekly time engaging in PA (in minutes) was calculated based on the reported time spent in leisure time sports including both community sports and unorganized sports, intra-curricular PA (PE and other weekly mandatory sports and PA), extracurricular school-based sports, and walking and cycling (not distinguishing between walking and cycling for active transportation or leisure time). Since all necessary data regarding each of the variables used in the analyses were obtained from all the participants, there were no missing data. The IGLS estimation method in MLwiN (version 2.26) was used to conduct the multilevel regression analyses. Multilevel modeling does not require stronger assumptions than ordinary modeling with regard to normality of the data [39]. Multilevel models therefore assume that the distribution at each level comes from a Gaussian distribution. A technique for graphically assessing this assumption for each residual is to plot a normal score plot [40]. The normal score plots showed that, for eleven out of twelve conditions (4 variables at 3 levels respectively), the data were normally distributed.

First, a three-level null model (school, class, pupil) or intercept-only model including only the dependent variable was estimated for PA (null model 1), autonomous motivation (null model 2), controlled motivation (null model 3), and amotivation (null model 4), respectively. These null models partitioned the total variance of the examined dependent variable into the between-pupil (Level 1), between-class (Level 2) and between-school (Level 3) variance and further served as a baseline with which explanatory models were compared.

In a second step, age and SES were inserted in the models as covariates. Furthermore, all three types of motivation were entered as additional covariates in the model for PA (model 1a). Controlled motivation and amotivation were entered as additional covariates in the model with autonomous motivation as a dependent variable (model 2a). Similar strategies were followed for the two other motivation-models [autonomous motivation and amotivation as covariates in the model for controlled motivation (model 3a); autonomous and controlled motivation as covariates in the model for amotivation (model 4a)]. To compare these models with their respective intercept-only models, likelihood ratio tests were conducted. If a likelihood ratio test was statistically significant, the model with covariates was considered to be a better fit than the intercept-only model [41].

In a third step, sex, extracurricular school-based sports participation and participation in community sports were included as predictors in each of the models (model 1b, model 2b, model 3b and model 4b). These predictors were entered in the models as dichotomous variables (sex: 0 = boy, 1 = girl; extracurricular school-based sports participation: 0 = no extracurricular school-based sports participation in the current school year, 1 = extracurricular school-based sports participation in the current school year; participation in community sports: 0 = no participation in community sports in the current school year, 1 = participation in community sports in the current school year). In the same model, we also explored the two-way interaction effects between each of these three predictors (sex × extracurricular school-based sports participation; sex × community sports participation; and extracurricular school-based sports participation × community sports participation) and the three-way interaction effect (sex × extracurricular school-based sports participation × community sports participation). To interpret significant interactions, the regression equations were repeated several times by changing the reference category to obtain coefficients for all combinations of the used variables (sex, extracurricular school-based sports participation and community sports participation). Here again likelihood ratio tests were conducted to determine if the models with predictors were better fits than the models only including the covariates.

In a final step, only statistically significant predictors ameliorating each of the models were retained, resulting in the most parsimonious and interpretable models. The most parsimonious models for PA, controlled motivation and amotivation therefore only included the covariates and the three main effects as predictor (model 1c, model 3c and model 4c). Since all the interaction-effects were significant in the model for autonomous motivation, all predictors were retained in the final model (model 2b).

The level of significance was for all statistical analyses defined as lower than 0.05.

## Results

### Descriptives for extracurricular school-based sports and community sports participation

Overall, the majority of the children (75.98%) reported participating in extracurricular school-based sports during the current school year. Almost one third of the children (31.87%) spent more than one hour per week on extracurricular school-based sports, while 43.54% engaged in extracurricular school-based sports for 10 to 60 minutes weekly, the remaining 25.00% of the children spent less than 10 minutes per week in extracurricular school-based sports. A significantly positive relation was found between extracurricular school-based sports participation and community sports participation [χ^2^(1) = 26.25, *p* < 0.001]. More than three-quarters (76.79%) of the extracurricular school-based sports participants and more than half (60.32%) of the non-participants were member of at least one sports club. Only a small group of children, namely 9.53% (*n* = 100) of the total sample (*N* = 1049), participated neither in extracurricular school-based sports nor in community sports, while 17.64% of the children reported participating in extracurricular school-based sports at least once a year but not in community sports (Table 1).

**Table 1:** Cross tabulation extracurricular school-based sports and community sports

		**No extracurricular school-based sports participation**	**Extracurricular school-based sports participation**	**Total**
**No community sports participation**	N	*100*	*185*	*285*
	% within community sports	35.09%	64.91%	100.00%
	% within extracurricular school-based sports	39.68 %	23.21%	27.17%
	% of total	9.53%	17.64%	27.17%
**Community sports participation**	N	*152*	*612*	*764*
	% within community sports	19.90%	80.10%	100.00%
	% within extracurricular school-based sports	60.32%	76.79%	72.83%
	% of total	14.49%	58.34%	72.83%
**Total**	N	*252*	*797*	*1049*
	% within community sports	24.02%	75.98%	100.00%
	% within extracurricular school-based sports	100.00%	100.00%	100.00%
	% of total	24.02%	75.98%	100.00%

Similarly, the majority of the children (72.83%) reported participating in community sports, and of this group 80.10% participated in extracurricular school-based sports also. Of the children not engaging in any community sports, 64.91% (*n* = 185) reported participating in extracurricular school-based sports in the current school year.

Participation in extracurricular school-based sports significantly differed by sex [χ^2^(1) = 33.73, *p* < 0.001]. As shown in Table 2, participation was reported by 82.77% of the boys (*n* = 466) and 68.11% of the girls (*n* = 331). More than one third of the boys (36.70%) and one quarter of the girls (25.08%) spent more than one hour per week on extracurricular school-based sports, while 43.35% of the boys and 43.81% of the girls engaged in extracurricular school-based sports for 10 to 60 minutes weekly, the remaining 19.96% of the boys and 31.12% of the girls spent less than 10 minutes per week in extracurricular school-based sports. Participation in community sports did not significantly differ by sex [χ^2^(1) = 0.94, *p* = 0.33]. Almost three-quarters of the boys (74.07%) and the girls (71.40%) reported participating in community sports (Table 3) with 29.45% of the participants spending less than two hours per week on community sports, 43.68% between two and six hours per week and 26.75% more than six hours per week. The group of children neither participating in extracurricular school-based sports nor community sports consisted of 40.00% boys and 60.00% girls.

**Table 2:** Cross tabulation extracurricular school-based sports and sex

		**No extracurricular school-based sports participation**	**Extracurricular school-based sports participation**	**Total**
**Boys**	N	*97*	*466*	*563*
	% within sex	17.23%	82.77%	100.00%
	% within extracurricular school-based sports	38.49%	58.47%	53.67%
	% of total	9.25%	44.42%	53.67%
**Girls**	N	*155*	*331*	*486*
	% within sex	31.89%	68.11%	100.00%
	% within extracurricular school-based sports	61.51%	41.53%	46.33%
	% of total	14.78%	31.55%	46.33%
**Total**	N	*252*	*797*	*1049*
	% within sex	24.02%	75.98%	100.00%
	% within extracurricular school-based sports	100.00%	100.00%	100.00%
	% of total	24.02%	75.98%	100,00%

**Table 3:** Cross tabulation community sports and sex

		**No community sports participation**	**Community sports participation**	**Total**
**Boys**	N	*146*	*417*	*563*
	% within boys	25.93%	74.07%	100.00%
	% within community sports	51.23%	54.58%	53.67%
	% of total	13.91%	39.75%	53.67%
**Girls**	N	*139*	*347*	*486*
	% within girls	28.60%	71.40%	100.00%
	% within community sports	48.77%	45.42%	46.33%
	% of total	13.25%	33.08%	46.33%
**Total**	N	*285*	*764*	*1049*
	% within sex	27.17%	72.83%	100.00%
	% within community sports	100.00%	100.00%	100.00%
	% of total	27.17%	72.83%	100.00%

### Sports offer in extracurricular school-based sports

In Flanders, as noted earlier, extracurricular school-based sports are organized directly after school hours, during lunch breaks and on Wednesday-afternoon. Overall, soccer and swimming were the most frequently offered sports (in 90% of the schools), followed by athletics (86.7%), netball (83.3%) and hockey (76.7%). Other popular sports were badminton (66.7%), dance, tennis, softball (46.7%) and basketball (43.3%). Some sports were only offered by a minority of the schools: rugby, volleyball, squash (33.3%), table tennis, handball, rope skipping, skating (30%), ring stick (26.7%), cycling (23.3%), korfball (20%), self-defense and martial arts (16.7%), wall climbing (6.7%), gymnastics and circus techniques (3.3%).

During after school hours, the content of the extracurricular school-based sports was completely determined by the supervising teacher and typically consisted of popular sports such as soccer, hockey and badminton, combined with an initial warm-up game (e.g. dodgeball, tag and ball tag). During lunch breaks, ball sports and dance were the most frequently offered activities, while on Wednesday afternoon the extracurricular school-based sports offer mostly consisted of once-a-year-offered sports activities like swimming, athletics (most often running races) or skating.

### Self-reported physical activity according to participation in extracurricular school-based sports

To examine if children participating in extracurricular school-based sports and/or community sports are more or less physically active than their non-participating peers, a three-level model was estimated for weekly-based PA. Variance at school-level was not significantly different from zero [3.1%, χ^2^(1) = 1.20, *p* = 0.273]. The random part of the null model revealed that the variances at class-level [6.5%, χ^2^(1) = 4.38, *p* = 0.036] and at pupil-level [90.4%, χ^2^(1) = 495.58, *p* < 0.001] were significantly different from zero, suggesting that both the class-environment and individual pupil level characteristics may have an influence on pupils’ PA. Age, SES, autonomous motivation, controlled motivation, and amotivation were entered as covariates in the predictor models (Model 1a). Both age [β = 50.92, *S.E.* = 19.31, χ^2^(1) = 6.96, *p* = 0.008] and autonomous motivation [β = 118.24, *S.E.* = 18.23, χ^2^(1) = 42.09, *p* < 0.001] were found to be significantly related to pupils’ PA-levels. Pupil’s sex, extracurricular school-based sports participation and community sports participation were then entered as predictors in the next step. As shown in Table 4, girls were significantly less physically active than boys [β = -135.45, *S.E.* = 22.89, χ^2^(1) = 35.03, *p* < 0.001].

**Table 4:** Relationship between sex, extracurricular school-based sports participation, community sports participation and weekly self-reported physical activity

**Weekly self-reported physical activity (Model 1)**				
**Parameter**	**Null model 1**	**Model 1a**	**Model 1b**	**Model 1c**
**Fixed part**	β (S.E.)	β (S.E.)	β (S.E.)	β (S.E.)
*Intercept*	581.04 (21.78)	588.48 (24.10)	446.73 (62.62)	499.99 (38.15)
Age		64.88 (19.48)***	52.60 (19.13)**	50.92 (19.31)**
SES (high)^a^		−15.65 (24.06)	−35.33 (23.35)	−36.61 (23.31)
Autonomous motivation		156.94 (17.57)***	117.70 (18.28)***	118.24 (18.23)***
Controlled motivation		15.05 (19.05)	4.59 (18.37)	5.95 (18.43)
Amotivation		34.64 (19.89)	27.01 (19.14)	24.67 (19.17)
Pupil sex (girl)^b^			−102.38 (74.18)	−135.45 (22.89)***
Extracurricular school-based sports participation (yes)^c^			187.22 (68.93)*	157.62 (28.54)***
Community sports participation (yes)^d^			85.47 (77.59)	57.07 (27.95)*
Girl x extracurricular school-based sports			59.36 (92.40)	
Girl x community sports			40.27 (95.77)	
Extracurricular school-based sports x community sports			11.23 (85.54)	
Girl x extracurricular school-based sports x community sports			−184.65 (114.12)	
**Random part**	σ^2^ (S.E.)	σ^2^ (S.E.)	σ^2^ (S.E.)	σ^2^ (S.E.)
School-level variance	3 712.82 (4 501.84)	5 858.29 (4 095.83)	4 360.46 (3 835.24)	4 343.75 (3 958.93)
Class-level variance	11 240.89 (5 295.85)*	7 318.49 (4 011.82)	8 408.89 (4 135.84)*	9 040.08 (4 321.37)*
Pupil-level variance	151 314.31 (6 796.73)*** 136 386.27 (6 125.50)*** 124 771.37 (5 604.52)*** 125 725.64 (5 647.62)***			
Deviance test model	15 545.97	15 434.61***	15 344.24***	15 353.79***
χ^2^ (df)		11.36 (5)	90.37 (12)	80.18 (8)

*Note*. Values in parentheses are standard errors.

*p < .05; **p < .01, ***p < .001.

Reference category = 0; ^a^0 = low and medium SES, 1 = high SES; ^b^0 = boy, 1 = girl; ^c^0 = no participation in extracurricular school-based sports in the current school year, 1 = participation in extracurricular school-based sports in the current school year; ^d^0 = no participation in community sports in the current school year, 1 = participation in community sports in the current school year.

Model 1b and 1c were both compared to model 1a to calculate χ^2^.

After controlling for different covariates (see Table 4), children participating in extracurricular school-based sports were found to be significantly more physically active than children not participating in extracurricular school-based sports [β = 157.62, *S.E*. = 28.54, χ^2^(1) = 30.50, *p* < 0.001] while children participating in community sports were significantly more physically active [β = 57.07, *S.E*. = 27.95, χ^2^(1) = 4.17, *p* = 0.041] than children not participating in community sports. As none of the interaction effects between sex, participation in extracurricular school-based sports and participation in community sports were statistically significant, these were not included in the final model.

### Motivation towards sports and participation in extracurricular school-based sports

#### Autonomous motivation

In the null model for autonomous motivation, only the variance at pupil-level was significantly different from zero [95.0%, χ^2^(1) = 496.44, *p* < 0.001], which implies that there was no significant between-school and between-class variance in pupils’ autonomous motivation towards sports. With regard to the included covariates (Table 5) controlled motivation and amotivation were, unlike age and SES, significantly related to pupils’ autonomous motivation. Higher scores on amotivation were related to lower scores on autonomous motivation [β = -0.55, *S.E.* = 0.03, χ^2^(1) = 395.72, *p* < 0.001], while higher scores on controlled motivation were related to higher scores on autonomous motivation [β = 0.26, *S.E.* = 0.03, χ^2^(1) = 73.76, *p* < 0.001].

**Table 5:** Relationship between sex, extracurricular school-based sports participation, community sports participation and autonomous motivation towards sports

	**Autonomous motivation towards sports (Model 2)**		
**Parameter**	**Null model 2**	**Model 2a**	**Model 2b**
**Fixed part**	β (S.E.)	β (S.E.)	β (S.E.)
*Intercept*	4.15 (0.04)	4.13 (0.03)	3.38 (0.10)
Age		0.03 (0.03)	0.03 (0.03)
SES (high)^a^		0.05 (0.04)	−0.02 (0.04)
Controlled motivation		0.30 (0.03)***	0.26 (0.03)***
Amotivation		−0.64 (0.03)***	−0.55 (0.03)***
Pupil sex (girl)^b^			0.32 (0.13)**
Extracurricular school-based sports participation (yes)^c^			0.58 (0.12)***
Community sports participation (yes)^d^			0.90 (0.13)***
Girl x extracurricular school-based sports			−0.55 (0.16)***
Girl x community sports			−0.45 (0.16)**
Extracurricular school-based sports x community sports			−0.49 (0.14)***
Girl x extracurricular school-based sports x community sports			0.58 (0.19)**
**Random part**	σ^2^ (S.E.)	σ^2^ (S.E.)	σ^2^ (S.E.)
School-level variance	0.02 (0.01)*	0.00 (0.01)	0.01 (0.01)
Class-level variance	0.01 (0.01)	0.01 (0.01)	0.01 (0.01)
Pupil-level variance	0.66 (0.03)***	0.43 (0.02)***	0.36 (0.02)***
Deviance test model	2 556.76	2 116.65***	1947.14***
Χ^2^ (df)		440.11 (4)	169.51 (11)

*Note*. Values in parentheses are standard errors.

*p < .05; **p < .01, ***p < .001.

Reference category = 0; ^a^0 = low and medium SES, 1 = high SES; ^b^0 = boy, 1 = girl; ^c^0 = no participation in extracurricular school-based sports in the current school year, 1 = participation in extracurricular school-based sports in the current school year; ^d^0 = no participation in community sports in the current school year, 1 = participation in community sports in the current school year.

As the three-way interaction effect between sex, participation in extracurricular school-based sports and participation in community sports was statistically significant, averages of autonomous motivation according to sex, participation in extracurricular school-based sports and participation in community sports were calculated for each possible combination by changing the reference categories of each of the involved variables. Table 6 shows that boys were in general more autonomously motivated to engage in sports than girls. Only within the group of children participating in community sports but not in extracurricular school-based sports, no significant differences were noted between boys and girls. For both boys and girls, participation in community sports was related to the highest levels of autonomous motivation. It can also be seen that boys who participate in extracurricular school-based sports but not in community sports were significantly more autonomously motivated than boys not engaging in either one of those forms of organized sports [β = 0.58, *S.E.* = 0.19, χ^2^(1) = 8.96, *p* = 0.003], whereas such differences were not noted among girls.

**Table 6:** Comparison of autonomous motivation towards sports between different groups

	***No community sports participation***		***Community sports participation***	
	**No extracurricular school-based sports participation**	**Extracurricular school-based sports participation**	**No extracurricular school-based sports participation**	**Extracurricular school-based sports participation**
	β (S.E.)	β (S.E.)	β (S.E.)	β (S.E.)
Boys	3.38 (0.10)^a^	3.97 (0.07)^b^	4.28 (0.09)^c^	4.38 (0.04)^c^
Girls	3.71 (0.08)^a#^	3.74 (0.07)^a#^	4.16 (0.07)^b^	4.28 (0.05)^b#^

A β is significantly different from another β if they have other superscript letters.

^#^indicates a significant sex difference.

#### Controlled motivation

The random parts of the null model for controlled motivation showed that the variances at both the class- and the pupil-level differ significantly from zero with the pupil-level variance [94.7%, χ^2^(1) = 496.43, *p* < 0.001] largely exceeding the class-level variance [5.2%, χ^2^(1) = 5.40, *p* = 0.020]. Of all the covariates that were inserted in the model, only age was not significantly related to controlled motivation (Table 7). Children with a high SES were overall less controlled motivated towards sports [β = -0.11, *S.E.* = 0.04, χ^2^(1) = 8.41, *p* = 0.04]. Pupils’ autonomous motivation [β = 0.26, *S.E.* = 0.03, χ^2^(1) = 79.68, p < 0.001] and their amotivation [β = 0.22, *S.E.* = 0.03, χ^2^(1) = 50.26, *p* < 0.001] were both positively associated with their controlled motivation. The interaction effects were not retained in the final model for controlled motivation since none of these interaction effects were statistically significant.

**Table 7:** Relationship between sex, extracurricular school-based sports participation, community sports participation and controlled motivation towards sports

	**Controlled motivation towards sports (Model 3)**			
**Parameter**	**Null model 3**	**Model 3a**	**Model 3b**	**Model 3c**
**Fixed part**	β (S.E.)	β (S.E.)	β (S.E.)	β (S.E.)
*Intercept*	1.94 (0.03)	2.00 (0.03)	2.01 (0.10)	2.08 (0.06)
Age		0.01 (0.03)	0.00 (0.03)	0.00 (0.03)
SES (high)^a^		−0.12 (0.04)**	−0.11 (0.04)**	−0.11 (0.04)**
Autonomous motivation		0.25 (0.03)***	0.26 (0.03)***	0.26 (0.03)***
Amotivation		0.23 (0.03)***	0.23 (0.03)***	0.22 (0.03)***
Pupil sex (girl)^b^			−0.04 (0.13)	−0.09 (0.04)*
Extracurricular school-based sports participation (yes)^c^			0.09 (0.12)	0.04 (0.05)
Community sports participation (yes)^d^			0.05 (0.13)	−0.10 (0.05)*
Girl x extracurricular school-based sports			0.04 (0.16)	
Girl x community sports			−0.12 (0.19)	
Extracurricular school-based sports x community sports			−0.12 (0.14)	
Girl x extracurricular school-based sports x community sports			0.03 (0.19)	
**Random part**	σ^2^ (S.E.)	σ^2^ (S.E.)	σ^2^ (S.E.)	σ^2^ (S.E.)
School-level variance	0.00 (0.00)	0.00 (0.00)	0.00 (0.00)	0.00 (0.00)
Class-level variance	0.02 (0.01)*	0.02 (0.01)*	0.02 (0.01)*	0.02 (0.01)*
Pupil-level variance	0.40 (0.02)***	0.36 (0.02)***	0.36 (0.02)***	0.36 (0.02)***
Deviance test model	2 045.87	1 946.34***	1 933.49	1 936.01
Χ^2^ (df)		99.53 (5)	12.85 (11)	10.33 (7)

*Note*. Values in parentheses are standard errors.

*p < .05; **p < .01, ***p < .001.

Reference category = 0; ^a^0 = low and medium SES, 1 = high SES; ^b^0 = boy, 1 = girl; ^c^0 = no participation in extracurricular school-based sports in the current school year, 1 = participation in extracurricular school-based sports in the current school year; ^d^0 = no participation in community sports in the current school year, 1 = participation in community sports in the current school year.

Model 3b and 3c were both compared to model 3a to calculate χ^2^.

As can be seen in Table 7, boys were overall more controlled motivated than girls [β = 0.09, *S.E.* = 0.04, χ^2^(1) = 5.1, *p* = 0.024], while children participating in community sports were less controlled motivated than the reference category [β = -0.10, *S.E.* = 0.05, χ^2^(1) = 4.21, *p =* 0.040]. Children participating versus not participating in extracurricular school-based sports did not significantly differ with regard to controlled motivation.

#### Amotivation

Similar to autonomous motivation, only the variance at pupil-level was significantly different from zero [99.1%, χ^2^(1) = 510.56, *p* < 0.001], implying a lack of between-school and between-class variance in pupils’ amotivation towards sports. Table 7 shows that autonomous motivation was negatively related to amotivation [β = -0.50, *S.E.* = 0.03, χ^2^(1) = 403.54, *p* < 0.001] while controlled motivation was positively related to amotivation [β = 0.20, *S.E.* = 0.03, χ^2^(1) = 49.49, *p* < 0.001] (Table 8). Since none of the interaction effects were statistically significant in the model for amotivation, they were not retained in the final model.

**Table 8:** Relationship between sex, extracurricular school-based sports participation, community sports participation and amotivation towards sports

	**Amotivation towards sports (Model 4)**			
**Parameter**	**Null model 4**	**Model 4a**	**Model 4b**	**Model 4c**
**Fixed part**	β (S.E.)	β (S.E.)	β (S.E.)	β (S.E.)
*Intercept*	1.38 (0.03)	1.40 (0.03)	1.49 (0.10)	1.47 (0.06)
Age		0.03 (0.03)	0.03 (0.03)	0.03 (0.03)
SES (high)^a^		−0.05 (0.04)	−0.05 (0.04)	−0.05 (0.04)
Autonomous motivation		−0.50 (0.02)***	−0.50 (0.03)***	−0.50 (0.03)***
Controlled motivation		0.21 (0.03)***	0.20 (0.03)***	0.20 (0.03)***
Pupil sex (girl)^b^			−0.08 (0.12)	−0.10 (0.04)**
Extracurricular school-based sports participation (yes)^c^			0.00 (0.11)	−0.02 (0.04)
Community sports participation (yes)^d^			−0.10 (0.13)	−0.01 (0.05)
Girl x extracurricular school-based sports			−0.16 (0.15)	
Girl x community sports			0.06 (0.15)	
Extracurricular school-based sports x community sports			0.04 (0.14)	
Girl x extracurricular school-based sports x community sports			0.11 (0.18)	
**Random part**	σ^2^ (S.E.)	σ^2^ (S.E.)	σ^2^ (S.E.)	σ^2^ (S.E.)
School-level variance	0.01 (0.01)	0.00 (0.00)	0.00 (0.00)	0.00 (0.00)
Class-level variance	0.00 (0.00)	0.00 (0.00)	0.00 (0.00)	0.00 (0.00)
Pupil-level variance	0.50 (0.02)***	0.34 (0.02)***	0.33 (0.02)***	0.34 (0.02)***
Deviance test model	2 265.68	1 848.46***	1 837.07	1 841.31
Χ^2^ (df)		417.22 (4)	11.39 (11)	7.15 (7)

*Note*. Values in parentheses are standard errors.

**p < .01, ***p < .001.

Reference category = 0; ^a^0 = low and medium SES, 1 = high SES; ^b^0 = boy, 1 = girl; ^c^0 = no participation in extracurricular school-based sports in the current school year, 1 = participation in extracurricular school-based sports in the current school year; ^d^0 = no participation in community sports in the current school year, 1 = participation in community sports in the current school year.

Model 4b and 4c were both compared to model 4a to calculate χ^2^.

Children participating versus not participating in extracurricular school-based sports did not significantly differ with regard to amotivation [β = -0.02, *S.E.* = 0.04, χ^2^(1) = 0.20, *p* = 0.65]. Similarly children participating in community sports did not, compared to children not participating in community sports, significantly differ in terms of amotivation [β = -0.01, *S.E.* = 0.05, χ^2^(1) = 0.03, *p* = 0.86]. There is, however, a significant difference between boys’ and girls’ amotivation towards sports with girls being significantly less amotivated than boys [β = -0.10, *S.E.* = 0.04, χ^2^(1) = 7.13, * =* 0.007].

## Discussion

In the present study extracurricular school-based sports participation was investigated as one possible vehicle to offer opportunities for children to be physically active. Children in the last two grades of elementary school were chosen as the target population of the present study, since this age coinciding with the transition from childhood to adolescence is typically characterized by a decrease in both PA and sports participation [28]. The results of the current study demonstrate that, if extracurricular school-based sports are offered in elementary school, an overall high participation rate in extracurricular school-based sports is found.

The results furthermore reveal that extracurricular school-based sports not only reach children who already participate in community sports, but also almost two third (64.91%) of the children who do not yet participate in community sports. This means that some pupils who do not participate in sports outside the school context, nonetheless engage in extracurricular school-based sports activities. In contrast to community sports, the vast majority of extracurricular school-based sports programs in Flanders are free of charge. It is possible that some children would like to participate in community sports, but do not get that opportunity due to financial constraints. Eime et al. [23,42] indicated that transportation issues might be another reason why children do not engage in community sports outside school. Participation in extracurricular school-based sports has the advantage that it does not require additional transportation as extracurricular school-based sports are organized at school or in its neighborhood at times when the children are already at school. The school environment is also a familiar setting in which children may consider the barrier to participate in sports as relatively low. Especially for the less sports talented children, the step towards more selective and competitive community sports may seem challenging. For this group of children, extracurricular school-based sports can be a medium to get them acquainted with different kinds of sports without being pressured to compete. In a long-term perspective, extracurricular school-based sports may then serve as a vehicle to community sports participation.

Despite the promising findings of the present study, it must be noted that in our sample, only schools with an extracurricular school-based sports offer were included. Research from the Flemish Policy Centre Culture, Youth and Sports (2010) [43] shows that only 26% of the Flemish elementary schools actually offer extracurricular school-based sports. As extracurricular school-based sports programs reach almost two third of the children who do not engage in community sports, it would seem important that effort is put into offering extracurricular school-based sports in more elementary schools.

A second aim of the present study was to assess whether extracurricular school-based sports participants were more physically active in daily life than children who do not participate in extracurricular school-based sports, while controlling for their participation in community sports. The results indicate that children who participate in extracurricular school-based sports are more physically active in daily life than their non-participating peers. This difference in PA attributed to participation in extracurricular school-based sports is even larger than the increase in PA due to participation in community sports. Furthermore, the average amount of weekly time spent in extracurricular school-based sports by the participants is only a fraction of the difference in weekly PA between extracurricular school-based sports participants and non-participants, suggesting that children participating in extracurricular school-based sports have an overall more active lifestyle than their counterparts, independent of whether they engage in in community sports or not. These results are in line with the findings of Sallis et al. [19] and Haerens et al. [20] who found that the provision of PA organized at school is positively related to children’s total PA-levels. It is possible that participation in extracurricular school-based sports encourages children to be physically active in leisure time but not necessarily to participate in organized community sports. More specifically, the acquaintance with new sports or skill improvement in familiar sports can stimulate children to engage in sports activities outside the school context, such as playing soccer with friends on a grass field in the neighborhood [44]. On the other hand, given the cross-sectional character of the study, it is also possible that children who are more generally inclined to be physically active in leisure time are more engaged in extracurricular school-based sports participation.

The third aim of the present study was to evaluate whether children who participate in extracurricular school-based sports, compared to those who do not, are better (i.e., more autonomously) or relatively worse (i.e., more controlled) motivated or amotivated to participate in sports in general. It was found that boys not engaging in community sports but attending extracurricular school-based sports programs are more autonomously motivated towards sports than boys not engaging in community nor extracurricular school-based sports. It is thus indeed possible that there exists a group of boys who are autonomously motivated towards sports, who found their way to extracurricular school-based sports but not to community sports. For this group of autonomously motivated boys who might not get the chance to participate in community sports, extracurricular school-based sports could serve as a perfect opportunity to play sports. On the other hand, it might also be the case that a number of boys, by participating in extracurricular school-based sports, develop more autonomous motivation towards sports. Earlier research by Hagger et al. [27] indicates that autonomous motives in a PE context are related to autonomous motives in a leisure-time context in secondary school children from diverse countries. In other words, children who are autonomously motivated to participate in PE are more autonomously motivated to participate in sports than children who are less or not motivated to participate in PE, suggesting that transfer across contexts can take place. Given its cross-sectional nature, the results of the present study might therefore also indicate that participation in extracurricular school-based sports stimulates the development of autonomous motivation towards sports because pupils start to value and enjoy activities through participating in it. However, this reasoning would only hold for boys and not for girls, as for girls levels of autonomous motivation were overall more alike across groups, independent of whether they engaged in extracurricular school-based sports or not.

The results further indicate that extracurricular school-based sports participation is not related to pupils’ controlled motivation or amotivation towards sports. The combination of children’s overall low levels of amotivation and high levels of autonomous motivation appear to confirm the generally positive attitude that children of this age group have towards sports.

### Practical implications

As children who do not engage in extracurricular school-based sports or in community sports have lower PA-levels and a significantly lower autonomous motivation towards sports, the question arises how these children can be stimulated and motivated to engage in sports or in PA more generally. One possibility would be to adapt extracurricular school-based sports programs to the needs of this specific target group so that these programs become more attractive and thus more accessible for this group of children. Another target group that requires special attention are girls since they have lower participation rates in extracurricular school-based sports than boys. Furthermore, girls are overall less physically active and, with the exception of the group that does not participate in extracurricular school-based sports or community sports who displayed low levels of autonomous motivation overall, they are less autonomously motivated towards sports than boys. The type of sports offered in extracurricular school-based sports programs might be one of the reasons why girls are less attracted to extracurricular school-based sports than boys. In most schools, much of the extracurricular school-based sports time is dedicated to traditional team sports (e.g., soccer, netball and hockey), which are typically more popular among boys than girls. This emphasis on traditional ball games is similar to the focus in the extracurricular school-based sports offer in schools in England and Wales [11] and the United States [45]. Even though swimming and athletics, which are considered attractive sports for both boys and girls, are part of most schools’ extracurricular school-based sports offer, they are often only once-a-year-offered activities and might therefore not contribute to continued extracurricular school-based sports participation. To attract more girls to participate in extracurricular school-based sports it might be effective to more frequently offer those sports that are popular among girls (e.g. swimming, dance, rope skipping, gymnastics and aerobics) [46-48].

From a practical point of view, it is also important to keep in mind that extracurricular school-based sports are just one of various potential mediums to motivate children to an active lifestyle. The positive relationship between participation in extracurricular school-based sports and overall PA-levels makes extracurricular school-based sports one of many promising settings to stimulate inactive children to a more active lifestyle.

### Strengths and limitations

Since the current study was cross-sectional, it is not possible to determine the direction of the relationship between extracurricular school-based sports participation on the one hand and PA and motivation towards sports on the other hand, which is a limitation of the present study. The question therefore remains if participation in extracurricular school-based sports triggers a more active lifestyle and a more autonomous motivation towards sports or if it is rather vice versa and children who already have high PA-levels and a high autonomous motivation to participate in sports are more attracted to extracurricular school-based sports than their peers. In this respect, it would be interesting to investigate the direction of these relationships by implementing extracurricular school-based sports in elementary schools not offering extracurricular school-based sports at the moment. Intervention studies based on such implementations or longitudinal studies could provide more clarity on the direction of the relationships.

Besides the cross-sectional design of the study, a potential limitation is the way pupils were classified with regard to extracurricular school-based sports participation. As participation in extracurricular school-based sports was questioned by a simple ‘yes’ or ‘no’-question, pupils only participating once in extracurricular school-based sports during the current school year were categorized as extracurricular school-based sports participants. Consequently, the overall high percentage of participants may give a slightly distorted view. A third limitation is the possibility of over- or underestimation of certain variables (e.g. the weekly PA), especially given the young age of the participants. However, all questionnaires used in the present study were validated for the target population and a researcher guided the questioning procedure.

The present study had also some considerable strengths. A first strength of the present study was the sample size (*N* = 1049), obtained from 60 different classes out of 30 diverse elementary schools, which were located in different areas (urban, suburban and rural). This relatively large sample allowed us to presume that the results of the current study are representative for all Flemish children of the last two grades of elementary school, attending schools with an extracurricular school-based sports offer.

Another strength was the procedure followed to collect data. All the questions were read out loud and children had the possibility to ask further explanation if questions were unclear. This method has undoubtedly contributed to a reliable data set without missing values.

Third, multilevel regression analyses were employed to analyze the hierarchical data. With regard to the frequency and the content of the offer, extracurricular school-based sports can vary at school-level. By using multilevel analyses, this variance was automatically taken into account.

## Conclusions

Extracurricular school-based sports programs are often seen as ideal channels to promote sports participation and PA among children who are not active in community sports. The results of the present study indicate that in elementary schools offering extracurricular school-based sports, many children engage in those activities. The extracurricular school-based sports programs attract not only children already engaging in community sports, but also children not yet participating in community sports.

## Abbreviations

BRPEQ: Behavioral regulations in physical education questionnaire; FPAQ: Flemish physical activity questionnaire; PA: Physical activity; PE: Physical education; SDT: Self-determination theory; SES: Socioeconomic status.

## Competing interests

The authors declare that they have no competing interests.

## Authors’ contributions

ADM contributed to the questionnaire design, data collection, data management, statistical analysis, interpretation of results and drafting the manuscript. NA contributed to the statistical analysis, interpretation of results and critical review of the manuscript. GC contributed to the study design, questionnaire design and critical review of the manuscript. IDB contributed to the study design and critical review of the manuscript. LH contributed to the study design, questionnaire design, statistical analysis, interpretation of results, and critical review of the manuscript. All authors have read and approved the final manuscript.

## Supplementary Material

Additional file 1 Methodology of the FPAQ validity study. Click here for file
