# Supplementary material for: Extracurricular school-based sports as a motivating vehicle for sports participation in youth: a cross-sectional study
Source: Int J Behav Nutr Phys Act. 2014 Apr 7;11:48. doi: 10.1186/1479-5868-11-48 (PMC4233643; doi:10.1186/1479-5868-11-48)
Supplement: Additional file 1 — Methodology of the FPAQ validity study. [file 1479-5868-11-48-S1.docx]

*Additional file 1*

To determine the validity of the questionnaire in the current study, we measured children’s PA levels by means of accelerometers in a convenience sample of 61 children who filled out the FPAQ.

As those children (61 children from 12 different classes) wore an ActiGraph GT3X accelerometer (Manufacturing Technologies Inc., Shalimar, FL) during seven consecutive days, we were able to investigate correlations between self-reported physical activity levels and accelerometer-measured activities. Similar to previous studies (32), significant positive correlations r=0.311; p=0.045) between both were found, further raising confidence in the validity of the questionnaire.

Children were instructed to wear the accelerometer on the right hip during waking hours and to remove it only for sleeping, water-based activities such as showering or swimming and activities that disallow the wearing of an accelerometer (e.g. defense sports). Participants had a logbook in which they were asked to write down the exact time periods during which and the reason why the accelerometer was removed. All the accelerometers were initialized to save data over 60 seconds epoch time intervals. The data in each epoch represent the intensity of the activity performed. In the present study, the cutoff points for physical activity in youth (6–16 years) as defined by Puyau, Adolph, Vohra, and Butte (2002) were used. (Registrations below 800 counts per minute were categorized as sedentary behavior, whereas registrations between 801 and 3199 counts per minute were considered light PA. Moderate PA included all count registrations between 3200 and 8199 per minute and vigorous PA all registrations above 8200 counts per minute). Consequently the time spent on sedentary behavior and PA of light, moderate, and vigorous intensity during a randomly chosen week was registered for each of the participants wearing an accelerometer. Sedentary behavior is defined by the Sedentary Behavior Research Network as any waking behavior characterized by an energy expenditure of ≤ 1.5 METs while in sitting or reclining posture (Sedentary Behaviour RN, 2012).

The activities during which the accelerometer was not worn but were registered in the logbook as activities with an energy expenditure greater than 3.0 METs were taken into account by calculating the number of minutes these activities were performed. Accelerometerdata were scored and interpreted using the MeterPlus software (version 4.3) from Santech, Inc.
